# 

**Published:** 1886-05

**Authors:** 


					﻿a cotoy.
MAY, 1886.
.F	.
$1.00 per year.
HALLS*
J<ai;\\i-
HEAITH
Established 1854
> V-t- 33
5
OFFICE, 75 & 77 BARCLAY STREET, NEW YORK.
Entered as Second-class Matter at the Post-Offlce, New York.
				

